# Factors Influencing the Efficacy of Anti-PD-1 Therapy in Chinese Patients with Advanced Melanoma

**DOI:** 10.1155/2019/6454989

**Published:** 2019-09-26

**Authors:** Lingdi Zhao, Yonghao Yang, Baozhen Ma, Wei Li, Tiepeng Li, Lu Han, Yong Zhang, Yi-Man Shang, Hongwei Lin, Quanli Gao

**Affiliations:** Department of Immunotherapy, Affiliated Cancer Hospital of Zhengzhou University & Henan Cancer Hospital, Zhengzhou City, Henan Province, China

## Abstract

**Purpose:**

Anti-PD-1 antibody improves the survival of patients with advanced melanoma. However, the efficacy and safety of anti-programmed death protein 1 (PD-1) antibody have not been fully elucidated in Chinese melanoma patients, who show high frequency of mucosal and acral melanoma subtypes; besides, the factors influencing the efficacy of anti-PD-1 antibody have not been evaluated broadly.

**Patients and Methods:**

Patients with advanced melanoma treated with regimens containing anti-PD-1 antibody from June 2016 to January 2019 were evaluated. Baseline characteristics and blood parameters were assessed, and outcome and adverse events were evaluated according to different regimens. The Cox proportional hazards regression model was used for univariate and multivariate analyses.

**Results:**

A total of 51 patients with advanced melanoma were included in this study. The overall objective response rate (ORR) was 17.6%, the disease control rate was 58.5%, and the median time to progression was 5.2 months. The ORR of patients with PD-1 blockade-based combination therapy, without liver metastases and higher level of C-reactive protein (CRP) before PD-1 blockade, is higher than that of those not. Univariate analysis based on clinical features showed that ECOG scores, liver metastasis, elevated lactate dehydrogenase (LDH), and CRP levels were the factors affecting time to progression (TTP). Multivariate analysis showed that elevated CRP before PD-1 blockade was an independent predictive factor for ORR of PD-1 blockade therapy (*P*=0.009), while only Eastern Cooperative Oncology Group (ECOG) score was an independent predictor for TTP (*P*=0.032). The treatment was well tolerated in these cohort patients, and there was no treatment-related death.

**Conclusion:**

Anti-PD-1 antibody-containing regimen was safe and effective in Chinese patients with advanced melanoma, and elevated CRP and ECOG score were independent factors predicting the efficacy of anti-PD-1 therapy.

## 1. Background

With an annual growth rate of 3–5%, melanoma has become one of the fastest growing tumors of all malignant tumors [1]. In 2018, 287,723 new cases of cutaneous melanoma occurred with 60,712 deaths worldwide [2]. In the United States, most melanoma cases occur on sites of sun-irradiated skin. Long-term chronic sun-irradiated injury may lead to increased mutations, and the tumor mutation burden is relatively high [3]. Before the emergence of immune-checkpoint inhibitors, the main therapeutic modality for metastatic melanoma was chemotherapy; the ORR was about 10%, and the median survival time was about 10 months [4]. The emergence of immune-checkpoint inhibitors such as anti-cytotoxic T lymphocyte antigen-4 (CTLA-4) and anti-PD-1 antibodies has changed the therapeutic modality for metastatic melanoma greatly, increasing the possibility of long-term survival for some patients. The ORR of anti-PD-1 antibodies alone is about 30% [5, 6]; in combination with anti-CTLA-4 antibodies, it could be as high as 57.6% [7].

Results from China Cancer Statistics in 2014 showed that the incidence of melanoma was 0.6/100,000 [8]. Melanoma in China has two distinctive features: younger age and a more advanced stage at diagnosis [8, 9], causing a great burden on patients' families and a high recurrence rate after resection. For patients with advanced melanoma, the efficacy of dacarbazine-based chemotherapy is only about 10%, and the median progression-free survival (PFS) is just about 2 months [4]. About 41.8% of melanomas in China occur at the extremities such as the feet, hands, and under the nails (acral melanoma), and about 22.6% of melanomas occur in the mucosa of the rectum, anus, vulva, mouth, and nasopharynx (mucosal melanoma) [10]. The incidence of *BRAF* mutations in Chinese melanoma patients is 25.5%, of which the most common mutation is *V600E*, accounting for 89.1% of mutations [9]. Acral and mucosal melanoma accounts for the majority of melanomas in China; however, acral and mucosal melanoma has obvious genetic and clinical features, low somatic mutation burden, poor response to treatment, and poor prognosis [11–13]. As a vast majority of clinical trials with anti-PD-1 antibodies for advanced melanomas were conducted outside of China, reports of anti-PD-1 antibodies for metastatic melanomas are few. Although some clinical trials were conducted in Chinese patients with advanced melanoma, the ORR was about 18–20% when anti-PD-1 was used as monotherapy and the ORR could be improved to 50% when anti-PD-1 was combined with axitinib [14–16]. However, the numbers of patients in these studies were small, and the performance status of patients in clinical study was relatively good. In routine clinical practice, the efficacy is usually not as good as that in clinical studies. Therefore, we performed this retrospective study to investigate the efficacy and safety of anti-PD-1 antibody-based therapy and to explore clinical factors that may influence the efficacy of anti-PD-1 therapy in Chinese patients with advanced melanoma.

## 2. Materials and Methods

### 2.1. Patients

Patients with pathologically diagnosed metastatic melanoma who underwent anti-PD-1-based therapy at the Department of Immunotherapy, Affiliated Cancer Hospital of Zhengzhou University & Henan Cancer Hospital (Zhengzhou, China), from June 2016 to January 2019 were enrolled in this retrospective study. The requirement for informed consent was waived due to the retrospective nature of this study. The study was approved by the Ethics Committee of Henan Cancer Hospital and was conducted in accordance with the principles expressed in the Declaration of Helsinki of 1975, revised in 2013. We reviewed the medical records of patients with metastatic melanoma who underwent anti-PD-1-based therapy using the hospital database. Inclusion criteria were as follows: (1) detailed medical histories collected and physical examinations performed; (2) complete blood cell counts and biochemical analyses performed; (3) received PD-1 blockade alone or in combination with other treatment (such as antiangiogenesis, interferon, tumor-infiltrating lymphocytes, or ipilimumab); and (4) expected lifespan of more than 3 months.

Clinical data for each patient, including gender, age, pretreatment complete blood cell count, lymphocyte subsets analysis, the status of *BRAF V600E*, PD-1 blockade agents, cycles of PD-1 blockade, LDH, CRP, albumin (ALB), efficacy, and survival time, were retrospectively reviewed.

### 2.2. Treatment and Follow-Up

All patients received anti-PD-1 antibody (either nivolumab or pembrolizumab); nivolumab was administered at a dose of 3 mg/kg every 2 weeks, and pembrolizumab was administered at a dose of 2 mg/kg every 3 weeks by intravenous infusion. For patients who got complete response (CR), another two doses of anti-PD-1 agent were conducted for consolidation therapy and then stopped. If the progressive disease (PD) was evaluated for the first time, then after another 4–6 weeks, another computed tomography (CT) scan was performed again, and if PD was evaluated again, then anti-PD-1 therapy was discontinued. Treatment was discontinued if the unacceptable toxicity appeared.

### 2.3. Efficacy and Safety Assessment

Radiological evaluation was performed at baseline and every 8 or 9 weeks. Responses were assessed according to Response Evaluation Criteria in Solid Tumors (RECIST) version 1.1; the assessment results included CR, partial response (PR), stable disease (SD), and PD. The ORR and disease control rate (DCR) were also calculated. TTP was defined from the date of treatment to disease progression or death. Overall survival (OS) was calculated from the initial treatment date to the date of death due to any reason. Patients who did not experience progression or were still alive at the last follow-up were censored. The severity of adverse events caused by treatment was monitored and graded according to the National Cancer Institute Common Terminology Criteria for Adverse Events (NCI-CTCAE version 4.0).

### 2.4. Statistics

Statistical Package for Social Sciences version 22.0 (SPSS Inc., Chicago, IL, USA) was used for data analyses. Neutrophil-to-lymphocyte ratio (NLR) was obtained by dividing the neutrophil count by the lymphocyte count. Platelet-to-lymphocyte ratio (PLR) was obtained by dividing the platelet count by the lymphocyte count. The receiver operating curve was used to determine the optimal cutoff values of pretreatment NLR and PLR which were defined as the points of maximum sensitivity and specificity. Dichotomous variables were expressed as means and standard deviations, and continuous variables were expressed as medians and ranges. The chi-square (*χ*^2^) test or *t*-test was used to describe the differences in demographic and clinical variables. The log-rank test was used to compare the survival curves. A Cox proportional hazards regression model was used for univariate and multivariate analyses. A two-sided probability value (*P* value) of less than 0.05 was considered significant.

## 3. Results

### 3.1. Defining the Cutoff Values of the Best NLR and PLR

**Figure 1:**
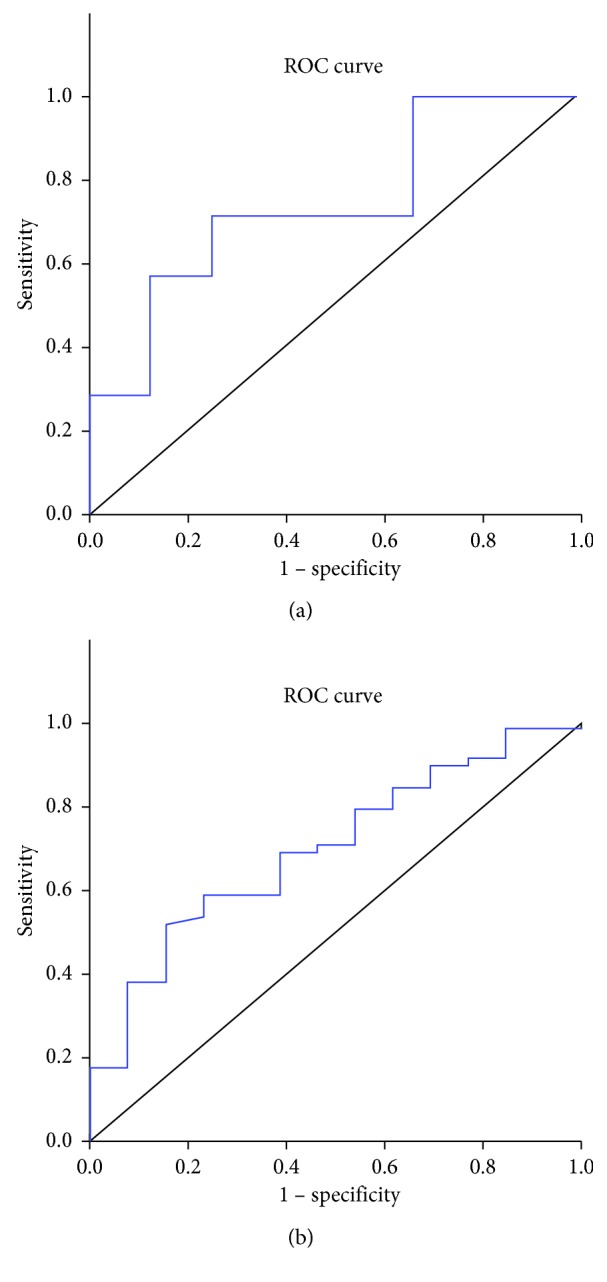
(a) The NLR ROC and the area under the curve was 0.738. (b) The PLR ROC and the area under the curve was 0.697.

According to receiver operating curve (ROC) analysis, the best cutoff value for NLR for operative prognosis was 2.3. Using this NLR cutoff value, the area under the curve was 0.738 (95% confidence interval: 0.516–0.96; *P*=0.049). Patients were then divided into either the low (<2.3; *n* = 29) or the high (≥2.3; *n* = 22) NLR groups. The best cutoff value for PLR for operative prognosis was 162.5. Using this PLR cutoff value, the area under the curve was 0.697 (95% confidence interval: 0.549–0.845; *P*=0.027).  Figure 1 shows the ROC curve for NLR and PLR.

### 3.2. Clinicopathological Characteristics at Baseline

Fifty-one patients (24 men and 27 women) were enrolled in this study, and the median age was 53.5 years (range, 28–81 years). As to the PD-1 blockade agents, 30 patients underwent nivolumab-based therapy and 21 underwent pembrolizumab-based therapy. Before the initiation of PD-1 blockade therapy, five patients had brain metastases and three underwent radiotherapy for the brain lesions, two of the three received brain radiotherapy got SD, and the other three got PD. Regarding the primary lesions, 16 (31.4%) were acral melanomas that arose from the soles, palms, and subungual sites, 17 (34.3%) were mucosal melanomas, and 18 (34.3%) were chronic sun-damaged (CSD) or non-CSD melanomas that arose in the skin other than in the acral sites (only one case in facial skin could be ascribed to CSD; the remaining cases were non-CSD). Thirty-eight patients received PD-1 blockade alone, and 13 patients received PD-1 blockade-based combination therapy. Baseline characteristics of the patients in the different treatment groups are summarized in Table 1. There were no statistical differences between the two groups except for LDH levels; more patients with high LDH levels underwent PD-1 blockade-based combination therapy. There were 13 patients who received PD-1 blockade-based therapy; the median age of these 13 patients was 51 years, and the median cycle of PD-1 blockade therapy was 6. Detailed short-term efficacy about the 13 patients who received PD-1 blockade-based therapy is described in [Supplementary-material supplementary-material-1].

### 3.3. Efficacy

#### 3.3.1. Objective Response Rate

Of the 51 patients, one achieved CR, eight PR, 21 SD, and 21 PD. The ORR was 17.6%, and the DCR was 58.8%. Of the 16 patients with acral melanomas, three achieved PR, seven SD, and six PD; the ORR was 18.8%, and the DCR was 62.5%. Of the 17 patients with mucosal melanomas, three achieved PR, seven SD, and seven PD; the ORR was 17.6%, and the DCR was 58.8%. Of the 18 patients with CSD or non-CSD melanomas, one achieved CR, two PR, seven SD, and eight PD; the ORR was 16.7%, and the DCR was 55.6%. ORR according to clinical features is described in detail in Table 2. Univariate analysis showed that liver metastases and normal serum CRP level were factors indicating lower ORR. Multivariate analysis showed that elevated CRP before PD-1 blockade was an independent predictive factor of the ORR of PD-1 blockade therapy (*P*=0.009). The efficacy results for different treatment groups are listed in Table 3.

#### 3.3.2. Time to Progression and Overall Survival

**Figure 2:**
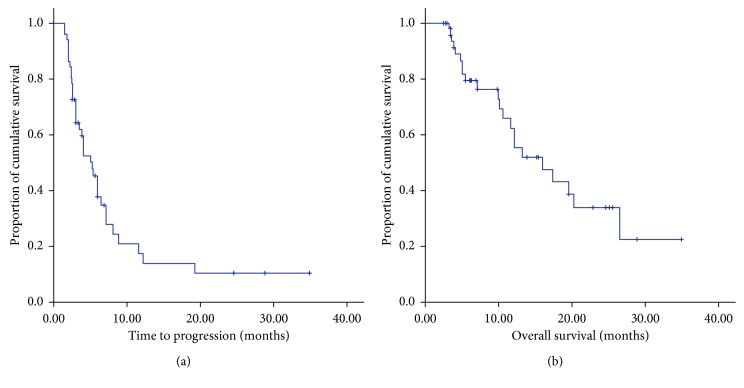
(a) TTP and (b) OS curve of all the 51 patients. The median TTP was 5.2 months, and the median OS was not achieved until the last follow-up.

The median TTP for all 51 patients was 5.2 months (95% confidence interval (CI): 3.7–6.7). The median TTP in the PD-1 blockade alone and PD-1 blockade-based combination groups was 5 months (95% CI: 3.4–6.6) and 7 months (95% CI: 2.9–12.8), respectively.  Figure 2 shows the TTP curve and OS curve. The median TTP in patients with acral melanomas, mucosal melanomas, and CSD/non-CSD melanomas was 5.3 months (95% CI: 2.4–8.2), 6.0 months (95% CI: 2.9–9.1), and 4 months (95% CI: 1.9–6.1), respectively. Detailed median TTPs according to clinical features are listed in [Supplementary-material supplementary-material-1]. Univariate analysis based on clinical features revealed that ECOG scores, liver metastasis, and elevated LDH and CRP levels were factors affecting TTP; multivariate analysis indicated that only the ECOG score was an independent predictor for shorter TTP (*P*=0.032).

### 3.4. Safety

The treatment was well tolerated by patients in this cohort. The incidence of adverse events (AEs) was 63.5% (33/52), most of which were grade 1-2. Grade 3-4 AEs were observed in 9.6% (5/52) of patients. Among the five patients with grade 3-4 toxicities, three had elevated transaminases, one exhibited grade 3 hypertension transiently, and one presented with grade 3 uveitis. The patient with hypertension developed elevated blood pressure about ten hours after the first and second dose of nivolumab which lasted for about 6 hours, and antihypertensive drugs were required; no hypertension occurred from the third dose of nivolumab. Treatments were delayed in the three patients with grade 3 elevated transaminases. PD-1 blockade therapy was permanently discontinued in the patient with grade 3 uveitis. It should be noted that grade 2 pneumonia developed in one patient; 2 months after the discontinuation of therapy, the pneumonia was downgraded to grade 1, and the patient continued PD-1 blockade therapy without any sign of deterioration of pneumonia. The occurrence of immune-related AEs was 68.5%. There were no treatment-related deaths. Treatment-related toxicities are listed in Table 4.

## 4. Discussion

This study showed that anti-PD-1 therapy is effective and safe in Chinese patients with advanced melanoma. The ORR was 17.6% (9/15), the DCR was 58.5% (30/51), and the median TTP was 5.2 months. These results are consistent with the results of previous studies that used nivolumab and pembrolizumab for advanced mucosal melanomas where the ORR was about 20% and the median PFS was approximately 3 months [17, 18]. Multivariate analysis identified elevated CRP as an independent predictive factor of the efficacy of PD-1 blockade therapy (*P*=0.009) and ECOG score as an independent predictor of a shorter TTP (*P*=0.032).

Further analysis revealed that other immunomodulatory therapies (such as interferon intratumoral injection, tumor-infiltrating lymphocyte transfusion, and anti-CTLA-4 antibodies in this study) could improve the efficacy of anti-PD-1 antibodies (*P*=0.023). Synergistic effects of antiangiogenesis and immunological checkpoint inhibitors have been confirmed in advanced non-small cell lung cancer, advanced renal cancer, and advanced hepatocarcinoma [19–21]. Synergy between anti-PD-1 therapy and antiangiogenesis may be explained by immune cells entering tumor tissues, normal vascular endothelium ensuring targeting of the tumor vasculature to enhance T-cell activity, and tumor angiogenesis helping tumor cells escape immune attack through cytokines such as vascular endothelial growth factor (VEGF), prostaglandin E2, interleukin- (IL-) 10, and local tumor hypoxia. At the same time, VEGF exerts immunosuppressive effects by inhibiting the adhesion of lymphocytes to activated endothelial cells and activated immunoregulatory cells (e.g., inhibiting the maturation of dendritic cells, inhibiting T-cell development and differentiation, and increasing inhibitory cells) [22]. Intratumoral injection of interferon could increase the expression of CXCL-10, CXCL-11, and CCL5 in tumors and help lymphocytes to localize in tumor sites to perform antitumor activities [23, 24]. Tumor cells could induce the expression of PD-1 molecules on lymphocytes which inhibits their antitumor activity. Moreover, tumor cells could inhibit the activity of lymphocytes through the PD-1/PD-L1 signaling pathway, thereby providing a theoretical basis for anti-PD-1 therapy plus intratumoral injection of interferon.

Performance status (PS) is the strongest prognostic factor of survival in patients with metastatic cancer. Patients with an ECOG PS 2 showed poor efficacy of anti-PD-1 therapy in this retrospective study regardless of age. Poor efficacy in patients with ECOG PS ≥ 2 is difficult to interpret. The literature includes minimal information on outcomes with chemotherapy or cancer immunotherapy, although Necchi et al. reported that OS was more than twofold worse in metastatic urothelial carcinoma patients with ECOG PS ≥ 2 compared with patients with ECOG PS 0 [25]. A poor PS represented decreased tolerability, poor response, and poor survival outcomes in this setting [26]. As there were more patients in the treatment-naive group with poor performance and received anti-PD-1 monotherapy, the mTTP and mOS of treatment-naïve patients were shorter in numerical value than those of pretreated patients.

Consistent with prior studies in which patients with solid tumors treated with anti-PD-1 antibodies with the presence of liver metastases have shown inferior response rates and PFS [27, 28], patients with liver metastases in this study showed poor efficacy regarding anti-PD-1 therapy. No CR or PR was achieved in any of the 15 patients with liver metastases. Moreover, the TTP of patients with liver metastases was shorter than that in patients without liver metastases. Liver metastasis may be correlated with elevated LDH level and poor prognosis, which may suggest poor efficacy of anti-PD-1 therapy [29].

CRP is an inflammatory factor, which can recognize changes in itself as well as exogenous molecules. This recognition leads to the production of a proinflammatory response signal that activates the acquired immune system, thus improving the defense function. CRP in serum is mainly synthesized in hepatocytes and regulated by IL-6, IL-1, and tumor necrosis factor (TNF). The presence of proinflammatory cytokines and TNFs in the tumor microenvironment is one of the causes of elevated serum CRP in patients with malignant tumors [30, 31]. CRP could also lead to excessive cell proliferation and subsequent DNA damage by promoting chronic inflammation [32] which might increase the mutation burden in local tumors and make the tumor more sensitive to anti-PD-1 therapy.

NLR and PLR, representing systemic inflammation [33], were reported to be correlated with the efficacy of immune-checkpoint inhibitors. However, we could not verify the correlation between NLR or PLR and the efficacy of anti-PD-1 therapy. The possible reasons for this inconsistency are the heterogeneity of the included patients and the limited number of cases.

LDH is a ubiquitous enzyme present in mammals, yeast, plants, and microorganisms. LDH plays a key role in the Warburg effect, and this metabolic pathway is prevalent in cancer cells independent of the presence of oxygen [34]. In many types of cancers, LDH is elevated and has been associated with tumor growth, maintenance, and invasion. LDH is regulated by hypoxia-inducible factors (HIFs) in cells [35]; the synthesis of HIF is increased in genetic mutations or hypoxia and it participates in tumor metastasis, angiogenesis, and glycolysis, leading to the expression of related proteins [35, 36]. Many studies have shown that elevated serum LDH is a poor prognostic factor in cancer patients [35, 37–39]. In this study, most of the patients with elevated LDH (11/16) underwent anti-PD-1-based combination therapy, and there were no statistical differences in ORR and TTP between the patients with normal serum LDH levels and those with elevated serum LDH levels, but there was a trend that the TTP and OS for the patients with normal LDH were longer than those with elevated LDH.

ALB is considered a nutritional index with the ability to stabilize DNA replication and cell growth, buffer various biochemical changes, and exert antioxidant effects against carcinogens [40]. Malnutrition, which is reflected by a low ALB level, could weaken defense mechanisms such as cellular and humoral immunity and phagocytic function, resulting in an increased possibility of infection and poor response to infection and anticancer treatment [41]. Potential mechanisms include malnutrition and an imbalanced tumor microenvironment. Only five patients with decreased ALB were included in this study; therefore, the ORRs between the two groups could not be compared directly. However, no remission was seen in these five patients which implied a low ORR in patients with decreased serum ALB.

This study has some limitations. First, the retrospective nature of the study may have introduced underlying bias and confounding factors. However, this was a single-center study that included all consecutive advanced melanoma patients treated with anti-PD-1 based therapy, thereby limiting the underlying selection bias inherent to this kind of study. Second, the number of patients included in this study was relatively small which limited its practicality. Third, the majority of subtypes in this study were mucosal, acral, and non-CSD melanomas which have different pathogeneses to skin melanoma that occurs in Americans (mainly CSD melanoma). Thus, the results of this study cannot be compared with the results of studies performed in American patients. However, our results were comparable to those reported by Wen et al. and Tang et al., which suggested the overall efficacy of anti-PD-1 antibodies in Chinese melanoma patients [14, 42]. Last, the expression of PD-L1 was only examined in a small number of patients, and the influence of PD-L1 expression on anti-PD-1 therapy was not analyzed.

In summary, the ORR of anti-PD-1 antibodies in Chinese patients with advanced melanoma was about 20%. Good efficacy of anti-PD-1 antibodies was observed in combination with other therapeutic modalities (such as antiangiogenesis, intratumoral interferon injection, and tumor-infiltrating lymphocytes) and in the presence of elevated serum CRP levels without liver metastasis. A higher serum CRP level was an independent predictor of a high response rate. Among the factors that influenced TTP (PS and levels of LDH and CRP), a good PS was an independent prognostic factor for a longer TTP. Prospective studies in a larger sample population are needed to further clarify the predictive value of these factors.

## Figures and Tables

**Table 1:** Baseline characteristics of the patients.

Characteristics	No. of patients (%)		
	PD-1 blockade alone	PD-1 blockade-based combination	*P* value
Gender			0.764
Male	17 (33.3%)	7 (13.7%)	
Female	21 (41.2%)	6 (11.8%)	
Age, mean (range)	56 (28–81)	51 (28–69)	0.212
ECOG status			0.176
0–1	26 (50.9%)	6 (11.8%)	
≥2	12 (23.5%)	7 (13.7%)	
Primary sites			0.979
Acral	12 (23.5%)	4 (7.8%)	
Mucosal	13 (25.5%)	4 (7.8%)	
CSD/non-CSD	13 (25.5%)	5 (9.8%)	
Metastatic sites			
Liver	9 (17.6%)	6 (11.8%)	0.176
Lung	16 (31.4%)	6 (11.8%)	0.611
Bone	9 (17.6%)	3 (5.9%)	0.864
Brain	5 (9.8%)	0 (0%)	0.153
Lymph nodes	25 (49.0%)	11 (21.6%)	0.16
LDH level			0.014
≤UNL	30 (58.8%)	5 (9.8%)	
>UNL	8 (15.7%)	8 (15.7%)	
CRP level			0.140
≤UNL	28 (54.9%)	6 (11.8%)	
>UNL	10 (19.6%)	7 (13.7%)	
ALB level			0.561
≥NLL	34 (66.7%)	12 (23.5%)	
<NLL	4 (7.8%)	1 (2.0%)	
NLR			0.574
≥2.3	16 (31.4%)	6 (11.8%)	
<2.3	22 (43.1%)	7 (13.7%)	
PLR			0.406
≥162.5	15 (29.4%)	7 (13.7%)	
<162.5	23 (45.1%)	6 (11.8%)	
*BRAF V600E* status			0.761
Mutation	9 (17.6%)	2 (3.9%)	
Wild-type	14 (27.5%)	6 (11.8%)	
Unknown	15 (29.4%)	5 (9.8%)	
PD-1 blockade agents			0.189
Nivolumab	24 (47.1%)	6 (11.8%)	
Pembrolizumab	14 (27.5%)	7 (13.7%)	
Treatment-naïve			0.961
Yes	22 (43.1%)	8 (15.7%)	
No	16 (31.4%)	5 (9.8%)	

ECOG: Eastern Cooperative Oncology Group; CSD: chronic sun-damaged; LDH: lactate dehydrogenase; CRP: C-reactive protein; UNL: upper normal limit; LNL: lower normal limit; ALB: albumin. All *P* values were two-tailed.

**Table 2:** Objective response rate according to clinical characteristics.

Clinical features	CR + PR (*n*)	SD + PD (*n*)	*χ* ^2^	*P* value
Gender				
Male	5	19	0.317	0.574
Female	4	23		
Age				
≥60 years	4	17	0.048	0.826
<60 years	5	25		
ECOG				
≥2	2	17	1.057	0.304
0–1	7	25		
Subtype				
Acral	3	13		
Mucosal	3	14	0.025	0.987
Non-CSD/CSD	3	15		
BRAF V600E				
Mutant-type	2	9		
Wild-type	4	16	0.175	0.916
Unknown	3	17		
PD-1 blockade				
Alone	4	34	5.201	0.023
Combination	5	8		
PD-1 blockade agent				
Nivolumab	5	25	0.048	0.826
Pembrolizumab	4	17		
Liver metastasis				
Yes	0	15	4.554	0.033
No	9	27		
Treatment-naïve				
Yes	7	23	1.621	0.203
No	2	19		
LDH level				
Normal	7	28	1.782	0.182
Elevated	2	14		
ALB level				
Normal	9	37	N/A	N/A
Lowered	0	5		
CRP level				
Normal	2	32	9.735	0.002
Elevated	7	10		
NLR				
≥2.3	6	16	2.741	0.140
<2.3	3	26		
PLR				
≥162.5	6	16	2.289	0.157
<165.5	3	26		

CR: complete remission; PR: partial remission; SD: stable disease; PD: progressive disease; ECOG: Eastern Cooperative Oncology Group; CSD: chronic sun-damaged; LDH: lactate dehydrogenase; CRP: C-reactive protein; ALB: albumin. All *P* values were two-tailed.

**Table 3:** Summary of responses data for different treatment groups.

Variables	PD-1 blockade alone	PD-1 blockade-based combination	*P* value
ORR	10.80%	35.70%	0.036
DCR	56.80%	64.30%	0.518
mTTP (months)	5.0	7.0	0.273
mOS (months)	13.0	NA^a^	0.242

ORR: objective response rate; DCR: disease control rate; TTP: time to progression; OS: overall survival; NA: not achieved. All *P* values were two-tailed. ^a^The median overall survival of the patients in this group was unavailable due to the limited follow-up time.

**Table 4:** Adverse events considered to be treatment related by investigators (NCI-CTC v4.0).

Adverse events	Number of patients with events (%)			
	PD-1 blockade alone (*n* = 38)		PD-1 blockade-based combination (*n* = 13)	
	Total	Grade 3-4	Total	Grade 3-4
Any	21 (55.3%)	1 (2.6%)	11 (84.6%)	4 (30.8%)
Elevated transaminase	5 (13.2%)	0 (0%)	5 (38.5%)	3 (23.1%)
Elevated bilirubin	1 (2.6%)	0 (0%)	3 (23.1%)	0 (0%)
Anemia	1 (2.6%)	0 (0%)	1 (7.7%)	0 (0%)
Leukopenia	2 (5.3%)	0 (0%)	1 (7.7%)	0 (0%)
Hypothyroidism	4 (10.5%)	0 (0%)	2 (15.4%)	0 (0%)
Hyperthyroidism	2 (5.3%)	0 (0%)	2 (15.4%)	0 (0%)
Elevated myocardial enzyme	1 (2.6%)	0 (0%)	3 (23.1%)	0 (0%)
Elevated creatinine	1 (2.6%)	0 (0%)	1 (7.7%)	0 (0%)
Pyrexia	2 (5.3%)	0 (0%)	4 (30.8%)	0 (0%)
Rash	1 (2.6%)	0 (0%)	1 (7.7%)	0 (0%)
Itchy skin	2 (5.3%)	0 (0%)	1 (7.7%)	0 (0%)
Fatigue	3 (7.9%)	0 (0%)	2 (15.4%)	0 (0%)
Vitiligo	1 (2.6%)	0 (0%)	1 (7.7%)	0 (0%)
Hypertension	1 (2.6%)	1 (2.6%)	4 (40.8%)	0 (0%)
Uveitis	0 (0%)	0 (0%)	1 (7.7%)	1 (7.7%)

## Data Availability

The data used to support the findings of this study are included within the article and the supplementary information.

## Supplementary Materials

Supplementary Materials Table S1: the detailed agents combined with PD-1 blockade in the combination group. Table S2: TTP and 9 estimated OS by clinical characteristics.
