# Supplementary material for: Factors Influencing the Efficacy of Anti-PD-1 Therapy in Chinese Patients with Advanced Melanoma
Source: J Oncol. 2019 Sep 26;2019:6454989. doi: 10.1155/2019/6454989 (PMC6791241; doi:10.1155/2019/6454989)
Supplement: Supplementary Materials — Table S1: the detailed agents combined with PD-1 blockade in the combination group. Table S2: TTP and 9 estimated OS by clinical characteristics. [file 6454989.f1.docx]

Supplementary table 1 The detailed agents combined with PD-1 blockade in the combination group

| The agents combined with PD-1 blockade | efficacy |
| --- | --- |
| Interferon intratumoral injection (n=5) | 2 PR, 2 SD, 1 PD |
| TIL intravenous infusion (n=1) | 1 CR |
| Apatinib (n=4) | 2 SD, 2 PD |
| Anlotinib (n=2) | 1 PR, 1 PD |
| Ipilimumab (n=1) | 1 PR |

Supplementary table 2 TTP and estimated OS by clinical characteristics

| characteristics | mTTP (months) | Estimated mOS (months) |
| --- | --- | --- |
| Gender Male | 5.2 (95%CI 3.2-7.2) | 17.2 (95%CI 9.0-25.4) |
| Female | 5.0 (95%CI 2.8-7.2) | 12.0 (95%CI 9.2-14.8) |
| ECOG 0-1 | 6.0 (95%CI 4.8-7.2) | 19.3 (95%CI 8.0-30.6) |
| ≥2 | 3.0 (95%CI 2.3-3.7) | 10.5 (95%CI 3.2-19.8) |
| BRAF wild-type | 6.0 (95%CI 3.7-8.3) | 19.3 (95%CI 13.4-25.2) |
| Mutant-type | 4.0 (95%CI 3.3-4.7) | 10.5 (95%CI 9.1-11.9) |
| unknown | 3.8 (95%CI 2.1-5.5) | 12.0 (95%CI 8.9-15.1) |
| Subtype Acral | 5.3 (95%CI 2.4-8.2) | 20.0 (95%CI 10.8-29.2) |
| Mucosal | 6.0 (95%CI 2.9-9.1) | 17.2 (95%CI 9.2-25.2) |
| Non-CSD | 4.0 (95%CI 1.9-6.1) | 12.0 (95%CI 9.9-14.1) |
| Liver metastasis Yes | 4.0 (95%CI 2.3-5.7) | 11.5 (95%CI 4.3-21.7) |
| No | 5.2 (95%CI 3.2-7.2) | 15.8 (95%CI 8.5-23.1) |
| LDH level Normal | 6.0 (95%CI 3.4-6.6) | 17.2 (95%CI 6.6-27.8) |
| Elevated | 3.0 (95%CI 2.1-3.9) | 12.0 (95%CI 2.9-23.4) |
| ALB level Normal | 5.2 (95%CI 3.6-6.8) | 15.8 (95%CI 8.2-23.4) |
| lowered | 3.0 (95%CI 0.5-5.5) | 3.4 (95%CI 2.3-6.7) |
| CRP level Normal | 3.8 (95%CI 2.7-4.9) | 12.0 (95%CI 9.2-14.8) |
| Elevated | 6.0 (95%CI 4.7-7.3) | 17.2 (95%CI 10.5-23.9) |
| NLR ≥2.3 | 4.0(95%CI 3.8-4.2) | 13.0(95%CI 8.8-17.2) |
| ＜2.3 | 6.0(95%CI 4.9-7.1) | 19.3(95%CI 6.1-32.5) |
| PLR ≥162.5 | 4.0(95%CI 1.4-6.6) | 15.8(95%CI 10.6-21.0) |
| ＜162.5 | 6.0(95%CI 4.1-7.9) | 17.2(95%CI 7.6-26.8) |
| Treatment-naïve Yes | 4.0 (95%CI 2.1-5.9) | 15.8 (95%CI 8.1-23.5) |
| No | 6.0 (95%CI 3.1-8.9) | 19.3 (95%CI 8.6-29.9) |
| Combination Yes | 5.0 (95%CI 3.4-6.6) | -- |
| No | 7.0(95%CI 2.9-12.8) | 13.0 (95%CI 6.7-19.3) |
